# Prolonged Peritoneal Attack: A Rare but Crucial Clinical Presentation of Familial Mediterranean Fever

**DOI:** 10.7759/cureus.69117

**Published:** 2024-09-10

**Authors:** Hirohisa Fujikawa, Takayuki Ando, Junichi Hirahashi

**Affiliations:** 1 Center for General Medicine Education, School of Medicine, Keio University, Tokyo, JPN

**Keywords:** familial mediterranean fever, periodic fever syndrome, prolonged abdominal pain, rare cause of acute abdominal pain, recurrent fever, serositis

## Abstract

Familial Mediterranean fever (FMF) presents with various symptoms. Episodic abdominal pain is one of the most prevalent clinical characteristics of FMF and usually improves within 24-48 hours. We encountered a 50-year-old male patient from Japan who experienced recurrent episodes (several episodes occurring per year) of abdominal pain with fever since his late 20s. The abdominal pain and fever began almost simultaneously in each episode. The abdominal pain typically lasted for 1-2 weeks, while the fever subsided within two days. He remained as immobile as possible because walking worsened the pain. MEditerranean FeVer (*MEFV*) gene analysis revealed exon 10 mutations (p.Met694Ile), resulting in an FMF diagnosis. Colchicine therapy effectively controlled the patient's FMF attacks. Although prolonged abdominal pain lasting over a week is an uncommon clinical characteristic of FMF, a proper diagnosis can improve the quality of life and prevent secondary amyloidosis. Therefore, clinicians should be aware of this rare clinical characteristic.

## Introduction

Periodic fever syndrome (PFS) is a group of innate immune system disorders characterized by recurrent episodes of fever and systemic inflammatory symptoms [1]. PFS includes familial Mediterranean fever (FMF), tumor necrosis factor receptor-associated PFS, cryopyrin-associated periodic syndrome, periodic fever, aphthous stomatitis, pharyngitis, and cervical adenitis syndrome [1]. Diagnosing PFS is challenging with conventional laboratory or imaging examinations; thus, it is frequently overlooked.

FMF is one of the most prevalent PFS disorders and is characterized by periodic episodes of fever and serositis. The most serious complication is the development of secondary amyloidosis [2]. FMF is an autosomal recessive disorder related to the MEditerranean FeVer (*MEFV*) gene, which encodes the protein pyrin that regulates the innate immune system [1]. FMF is most prevalent among Jewish, Armenian, Turkish, and Arab populations but is known to occur globally despite its name [3]. Each attack usually lasts for 24-72 hours and resolves spontaneously [4]. FMF is diagnosed based on patient history, inflammatory markers, and more recently, genetic testing [4]. Some clinical diagnostic criteria (e.g., Tel-Hashomer criteria) have been proposed [4]. Colchicine is primarily effective as a preventive treatment [4]. Biologics, such as interleukin-1 inhibitors and interleukin-6 inhibitors, are available for patients with colchicine-resistant or colchicine-intolerant FMF [4]. Early diagnosis of FMF is crucial because a delayed diagnosis can decrease the quality of life and lead to secondary amyloidosis.

Here, we report a case of a middle-aged male patient from Japan with rare FMF presentations in which abdominal pain attacks lasted over a week and were not properly diagnosed for more than 20 years.

## Case presentation

A 50-year-old male patient from Japan presented to our hospital with a one-day history of severe abdominal pain. A detailed history revealed several episodes per year of acute abdominal pain with fever since his late 20s. The abdominal pain and fever began almost simultaneously in each episode; however, the abdominal pain lasted for 7-14 days, while the fever subsided within two days. He remained as immobile as possible during these episodes because walking increased the pain. He had no symptoms other than abdominal pain and fever during all the attacks. Despite visiting more than 10 hospitals, none of the physicians had made a proper diagnosis before this attack. Only symptomatic treatment had been provided, resulting in a significant decline in his quality of life. He denied having any other medical history and PFS-related family history. He also denied taking any medication at the time of his visit to our hospital.

Examination revealed that the patient was ill-appearing and mildly febrile with a body temperature of 37.9ºC. Vital signs other than body temperature were unremarkable. The abdomen was soft but exhibited tenderness and mild percussion tenderness throughout. Laboratory examinations indicated a white blood cell count of 11.6 × 10^9^/L with a neutrophil percentage of 72.0% and a C-reactive protein level of 27.4 mg/L (Table 1). Contrast-enhanced abdomen-pelvis CT revealed no abnormalities. PFS, including FMF, was suspected. After discussing with the patient, a decision was made to follow up closely in an outpatient clinic rather than prescribe any medication.

**Table 1:** Laboratory tests.

	Test value	Normal range
WBC count	11.6 × 10^9^/L	3.3-8.6 × 10^9^/L
Hemoglobin	133 g/L	137-168 g/L
Platelet count	185 × 10^9^/L	158-348 × 10^9^/L
Aspartate transaminase	16 U/L	13-30 U/L
Alanine transaminase	12 U/L	10-42 U/L
Alkaline phosphatase	53 U/L	38-113 U/L
Gamma-glutamyl transferase	24 U/L	13-64 U/L
Total bilirubin	18.8 micromol/L	6.8-25.7 micromol/L
Lactate dehydrogenase	180 U/L	124-222 U/L
Creatine kinase	66 U/L	59-248 U/L
Urea nitrogen	5.2 mmol/L	2.9-7.1 mmol/L
Creatinine	75.1 micromol/L	57.5-94.6 micromol/L
Total protein	69 g/L	66-81 g/L
Albumin	42 g/L	41-51 g/L
Sodium	142.1 mmol/L	138-145 mmol/L
Potassium	3.7 mmol/L	3.6-4.8 mmol/L
Chloride	105 mmol/L	101-108 mmol/L
Glucose	5.2 mmol/L	4.0-6.0 mmol/L
C-reactive protein	27.4 mg/L	0-1.4 mg/L

The patient’s temperature improved immediately after the first outpatient visit, but the abdominal pain continued for approximately a week, similar to his usual attacks. We considered FMF as one of the differential diagnoses, given the recurrent episodes of abdominal pain and transient fever. We performed genetic testing with informed consent to confirm the diagnosis, considering the longer duration of abdominal pain than is typical for FMF cases and the absence of family history. *MEFV* gene testing revealed exon 10 mutations (p.Met694Ile), which confirmed the FMF diagnosis. Episodes of fever and abdominal pain completely disappeared after initiating colchicine therapy (1.0 mg/day). The patient has had no recurrence of the symptoms after ten months of follow-up.

## Discussion

This case involves a patient from Japan with repeated attacks of 7-14 days of abdominal pain accompanied by short-term fever, who was finally diagnosed with FMF. This is a cautionary case for clinicians, demonstrating that FMF can present with prolonged abdominal pain, leading to delayed diagnosis.

FMF attacks usually start from early childhood. A previous survey revealed that 65% developed FMF before the age of 10 and 80%-90% before the age of 20 [5]. However, making an accurate diagnosis often takes longer due to the wide range of FMF's clinical manifestations. A recent European cohort study found that the duration from onset to diagnosis was over 10 years in approximately 20% of patients with FMF [6]. FMF decreases the patient’s quality of life [7] and can cause secondary amyloidosis; thus, early diagnosis and intervention are necessary.

Patients with FMF present with varying symptoms, making diagnosis challenging [8]. For example, protracted febrile myalgia is an uncommon FMF presentation characterized by prolonged fever and severe generalized myalgia [9]. Massive chronic ascites is also a rare clinical presentation of FMF [10].

Episodic abdominal pain is one of the most prevalent presentations in patients with FMF. Moreover, 90% of the patients complain of abdominal pain [2], caused by peritoneal inflammation. Abdominal pain attacks usually start localized and then progress to more generalized pain, typically resolving without sequelae within 1-2 days [2, 8].

Conversely, prolonged abdominal pain attacks of more than one week are very rare in FMF. To the best of our knowledge, only a few cases of patients with FMF having such clinical presentations have been reported. Here, we present a review of the literature (Table 2) [11, 12]. Clinical progress was good in all cases after appropriate therapy for FMF was initiated. These cases emphasize the importance of recognizing FMF with a rare clinical course of prolonged abdominal pain, and that a proper diagnosis can significantly improve the patient’s quality of life.

**Table 2:** A review of the literature on cases of familial Mediterranean fever with prolonged abdominal pain (>1 week). References: Radisic M et al. [11] and Matsumoto H et al. [12]

Case no.	1	2	Our case
Author, year	Radisic M et al. (2006)	Matsumoto H et al. (2021)	Not applicable
Reference number	11	12	Not applicable
Age	26	46	50
Sex	Male	Male	Male
Origin	Argentine	Japanese	Japanese
MEFV	Genetic analysis was not performed	No *MEFV* mutation or polymorphisms	p.Met694Ile
Duration of abdominal pain	12 days	> 1 week	7-14 days
Symptoms other than abdominal pain	Fever, diarrhea, arthritis, myalgias	Fever	Fever
Time from onset to diagnosis	19 years	3 years	> 20 years
Treatment	Colchicine 1.0 mg/day	Colchicine 1.5 mg/day ---> Canakinumab 150 mg every 4 weeks	Colchicine 1.0 mg/day
Prognosis after therapy	No attacks	No attacks	No attacks

## Conclusions

Herein, we report a case of FMF with a prolonged peritoneal attack lasting 1-2 weeks. Despite the longer duration of abdominal pain than typical for FMF cases and the absence of a family history, *MEFV* gene testing confirmed the diagnosis of FMF. Our case is insightful because it suggests that FMF can lead to prolonged abdominal pain lasting more than one week. A prolonged abdominal pain attack is a very rare clinical presentation of FMF and has been under-recognized, with only two cases reported in the literature to date. However, proper diagnosis and therapeutic intervention would improve the patient's quality of life and help prevent secondary amyloidosis. Therefore, clinicians should be aware of this rare clinical presentation.
